# Skin Protective Activity of Silymarin and its Flavonolignans

**DOI:** 10.3390/molecules24061022

**Published:** 2019-03-14

**Authors:** Jitka Vostálová, Eva Tinková, David Biedermann, Pavel Kosina, Jitka Ulrichová, Alena Rajnochová Svobodová

**Affiliations:** 1 Department of Medical Chemistry and Biochemistry, Faculty of Medicine and Dentistry, Palacký University, Hněvotínská 3, 775 15 Olomouc, Czech Republic; jitka.vostalova@upol.cz (J.V.); e.tinkova@seznam.cz (E.T.); pavel.kosina@upol.cz (P.K.); jitka.ulrichova@upol.cz (J.U.); 2 Laboratory of Biotransformation, Institute of Microbiology, Academy of Sciences of the Czech Republic, Vídeňská 1083, 142 20 Prague, Czech Republic; biedermann@biomed.cas.cz

**Keywords:** *Silybum marianum*, collagenase, elastase, sun protection factor

## Abstract

*Silybum marianum* (L.) is a medicinal plant traditionally used in treatment of liver disorders. In last decades, silymarin (SM), a standardized extract from *S. marianum* seeds has been studied for its dermatological application, namely for UVB-protective properties. However, information on SM and its polyphenols effect on activity of enzymes participating in the (photo)aging process is limited. Therefore, evaluation of SM and its flavonolignans potential to inhibit collagenase, elastase, and hyaluronidase in tube tests was the goal of this study. The antioxidant and UV screening properties of SM and its flavonolignans silybin, isosilybin, silydianin, silychristin and 2,3-dehydrosilybin (DHSB) were also evaluated by a DPPH assay and spectrophotometrical measurement. DHSB showed the highest ability to scavenge DPPH radical and also revealed the highest UVA protection factor (PF-UVA) that corresponds with its absorption spectrum. SM and studied flavonolignans were found to exhibit anti-collagenase and anti-elastase activity. The most potent flavonolignan was DHSB. None of studied flavonolignans or SM showed anti-hyaluronidase activity. Our results suggest that SM and its flavonolignans may be useful agents for skin protection against the harmful effects of full-spectrum solar radiation including slowing down skin (photo)aging.

## 1. Introduction

The aging of skin is a complex progressive process leading to functional and aesthetic changes in skin tissue due to the effects of various intrinsic (genetic) and extrinsic (environmental) factors [1]. One of the most important extrinsic factors is solar radiation. UV (295–400 nm) wavelengths reaching the earth surface stimulate a complex reaction in skin, mainly the generation of reactive oxygen species (ROS). ROS oxidatively modify proteins and lipids to form carbonylated proteins and lipid peroxides, respectively, and activate enzymes (collagenase, elastase, hyaluronidase) that remodel components of the extracellular matrix (ECM) such as collagen, elastin, and hyaluronic acid. The activation of neutrophils is accompanied by the release of calcium-dependent zinc-containing matrix metalloproteinases (MMP), collagenase, and elastase into the extracellular space, resulting in breaking down collagen and elastic fibers [2]. Hyaluronidases degrade hyaluronic acid (HA) into smaller HA fragments of various lengths. This degradation is accompanied by a lowering of skin hydration and increase in the pro-inflammatory and pro-angiogenic properties of HA [3]. All of the above-mentioned enzymes contribute to skin elasticity decrease and wrinkle formation [4]. 

Phenolic compounds, due to their antioxidant properties, seem to be suitable candidates to diminish the above-mentioned structural and functional changes in skin. Milk thistle (*Silybum marianum* (L.) Gaertn. (Asteraceae)) is one of the oldest known herbal plants. It has been widely used in traditional European medicine for over two thousand years, especially for treating liver disorders. Silymarin (SM) is a standardized extract from *S. marianum* seeds that is rich in polyphenols. SM composition depends to a large degree on the plant variety, soil and weather conditions, and the time of harvest. Industrial preparations are only standardized for the total amount of silybin (SB), with the rest of the compounds ignored. Therefore, it is important to analyze the SM used and provide the composition [5]. SM is traditionally used as a hepatoprotective agent for its potent regenerative properties. Lately, SM is also utilized in dermatological and cosmetic preparations for its antioxidant effect and well-described ability to reduce UVB- and chemically-induced damage that may result in skin carcinogenesis [6]. SM contains flavonolignans (70–80%) and a chemically undefined fraction of polymeric and oxidized polyphenolic compounds (20–30%). The main active component in SM is the flavonolignan SB (mixture of diastereoisomers A and B). The less abundant compounds include the flavonolignans isosilybin (ISB, mixture of diastereoisomers A and B), silydianin (SD), silychristin (SC, mixture of diastereoisomers A and B) and trace amount of 2,3-dehydrosilybin (DHSB) and the flavonoid taxifolin. Due to their antioxidant, anti-inflammatory and immunomodulatory properties and ability to modulate various signaling pathways, SM and SB have been intensively studied for their potential in the prevention and/or slowing down of the progression of degenerative diseases [6,7].

**Figure 1:**
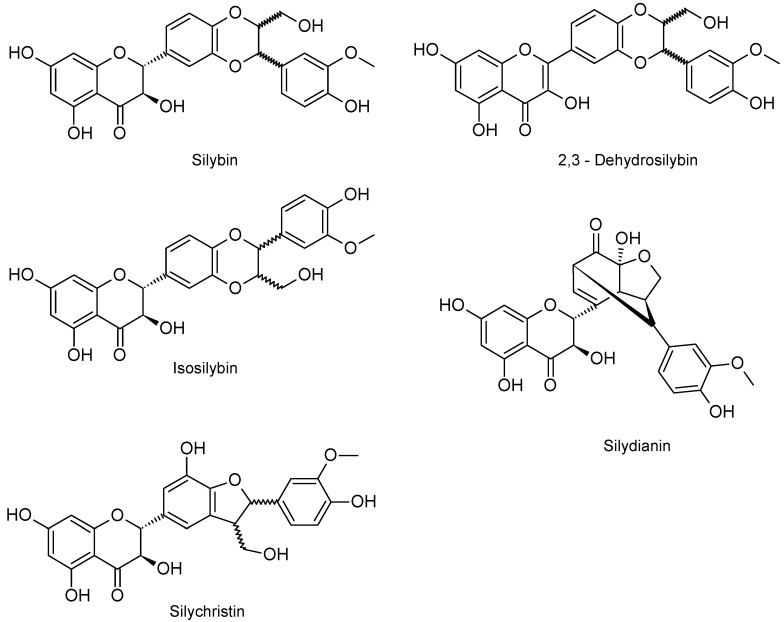
Structure of silymarin´s flavonolignans.

The aim of this study was to evaluate the effect of SM and its pure flavonolignans (SB, DHSB, SD, ISB and SC; Figure 1) on collagenase, elastase and hyaluronidase activity and compare their UV screening and radical scavenging properties. 

## 2. Results

### 2.1. Chemical Composition of Silymarin

The identification and quantification of polyphenolic constituents in the SM used was done by HPLC-MS. The content of individual flavonolignans and the flavonoid taxifolin in the SM is shown in Table 1. Total amount of flavonolignans was 67.6% and sum of flavonolignans including flavonoid taxifolin (TA) was 69.7%. SB (37.98%) is the most abundant component of SM. The content of the diastereomer SB B (21.64%) was higher than that of SB A (16.34%). The diastereomers of ISB were in proportions 2:1. The content of the diastereomer SC A was higher than that of SC B (7.5:1). Dehydro-derivatives DHSB (0.33%) and dehydrosilychristin (0.56%) were identified in minor amounts. 

For further inhibitory studies, SB (mixture of A and B diastereomers; ca. 1:1), ISB (mixture of A and B diastereomers; 95:5), SC (mixture of A and B diastereomers; 9:1), SD, DHSB and SM were used. 

### 2.2. DPPH Scavenging Activity

The scavenging ability of SM and the studied flavonolignans was evaluated by commonly used DPPH assay [8]. The minor constituent of SM, DHSB was the most active compound and its IC_50_ was 12.60 ± 0.84 mg/L. The ability of the other flavonolignans was in the order: DHSB > SC > SD >> ISB > SB. SM was much more effective (10-times) than its main constituent SB, the poorest flavonolignan tested. SM’s ability to scavenge DPPH radicals was comparable with that of SD. All flavonolignans including SM were less effective than reference compound quercetin (QE). The IC_50_ values of SM, the individual flavonolignans and QE are summarized in Table 2. 

### 2.3. UV Absorption Ability

**Figure 2:**
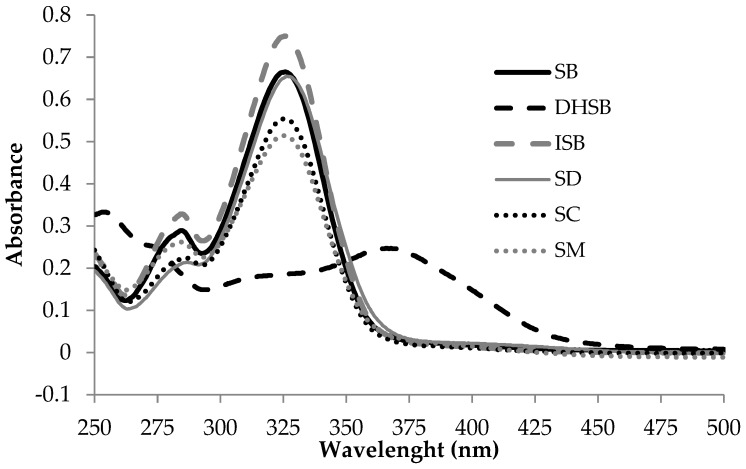
Absorption spectra of silymarin and flavonolignans (12.05 mg/L in 0.5% DMSO in PBS pH 7.5).

To study the ability of the studied compounds to absorb UV radiation and thus directly protect skin against solar radiation, their spectra were analyzed. All studied compounds including SM absorb UVB and UVA rays of solar radiation between 295 and 400 nm. SM and all flavonolignans except DHSB have quite similar absorption spectra and absorb UVB and mainly the shorter UVA wavelengths with the peak around 325 nm. In contrast, DHSB absorbs longer UVA wavelengths with the peak around 370 nm (Figure 2).

### 2.4. Sun and UVA Protection Factors

The SPF_(290–320)_, SPF_(290–400)_ and PF-UVA factors for SM and its constituents were determined by in vitro methods. The factors of pure compounds were determined in ethanolic solutions at the concentration of 50 µmol/L. As SM is a mixture of compounds, SM solution with the same mass concentration as flavonolignans (24.1 mg/L~50 µmol/L) was used. The SPF_(290–320)_, SPF_(290–400)_ and PF-UVA values are presented in Table 3. SM and all flavonolignans were less effective than ferulic acid (FA), used as a standard compound. The highest SPF_(290–320)_ and PF-UVA value was found for SB and DHSB, respectively.

### 2.5. Effect on Enzymes Activity

The modulation of the activity of collagenase, elastase and hyaluronidase by flavonolignans and SM was assessed by in vitro spectrophotometric and spectrofluorometric methods. The effectiveness of the studied compounds was evaluated in the concentration range of 0.05–100 mg/L (corresponding to a pure flavonolignans range of 0.1–200 µM). The concentration range used in individual inhibition studies depended on the solubility of the studied compounds in the reaction mixtures.

**Figure 3:**
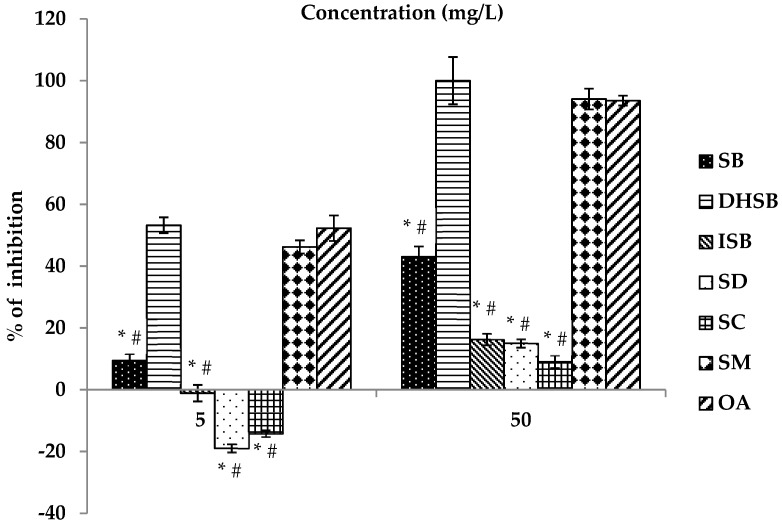
Anti-elastase activity of silymarin and flavonolignans. Silybin (SB), dehydrosilybin (DHSB), isosilybin (ISB), silydianin (SD), silychristin (SC), silymarin (SM), oleanolic acid (OA). OA is a standard inhibitor of elastase. Data are expressed as mean ± SEM from four independent experiments carried out in triplicate. ^#^ Significantly different from OA as the standard inhibitor at *p* = 0.05; * significantly different from SM at *p* = 0.05.

The effect of the studied flavonolignans (SB, DHSB, ISB, SD and SC) and SM on the activity of human leukocyte elastase is shown in Figure 3 and Table 4. DHSB and SB were the most effective flavonolignans. Other pure SM components (SC, SD and ISB) were markedly less effective than DHSB and SB. At the higher tested concentration of 50 mg/L (100 µM) the ability of SC, SD and ISB to inhibit elastase activity was less than 20% (see Figure 3). IC_50_ values could not be evaluated due to their limited solubility and effectiveness. The ability of DHSB to modulate the activity of human leukocyte elastase was comparable to the standard inhibitor oleanolic acid (OA). SM, the polyphenolic fraction from *S. marianum* seeds, had a similar dose-dependent effectiveness to the pure DHSB. 

**Figure 4:**
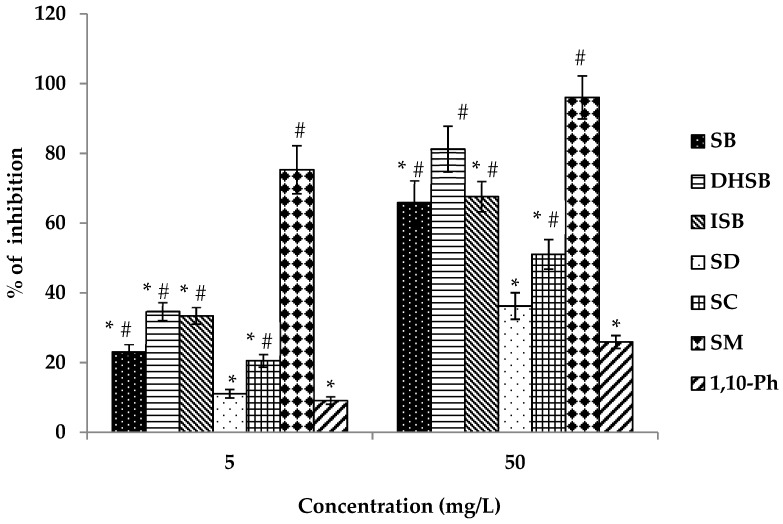
Anti-collagenase activity of silymarin and flavonolignans. Silybin (SB), dehydrosilybin (DHSB), isosilybin (ISB), silydianin (SD), silychristin (SC), silymarin (SM), 1,10-phenantroline (1,10-Ph). 1,10-Ph is a specific inhibitor of collagenase. Data are expressed as mean ± SEM from four independent experiments carried out in triplicate. ^#^ Significantly different from 1,10-Ph as the standard inhibitor at *p* = 0.05; * significantly different from silymarin at *p* = 0.05.

All studied flavonolignans and SM affected *Clostridium histolyticum* collagenase activity, see Figure 4 and Table 4. The most active flavonolignans were SB, DHSB and ISB. SD and SC were less potent than the above-mentioned compounds. All flavonolignans were more effective than the standard inhibitor 1,10-phenantroline (1,10-Ph). SM exhibited the strongest collagenase inhibition that was more pronounced at a concentration of 5 mg/L (75%) (see Figure 4).

SM and flavonolignans were also tested for their effect on the activity of hyaluronidase, however none of them modulated the enzyme’s activity (Data not shown).

## 3. Discussion

For more than thirty years, SM and its major flavonolignan SB have attracted attention in terms of their dermatological application due to their well-described UVB-photoprotective properties [10]. However, the majority of solar UV radiation is UVA radiation. Compared to UVB (280–415 nm), UVA light penetrates deep into the dermal layer of the skin. Dermal fibroblasts together with ECM components (elastin, collagen, hyaluronic acid) and vessels are the main target of UVA photons. Acute and chronic skin exposure to UV radiation is connected with the overproduction of ROS resulting in oxidative stress, activation of redox-sensitive signaling pathways and of MMP (collagenases, elastase), the degradation and oxidative modification of ECM components and accumulation of oxidatively modified products. All these effects of solar UV radiation are involved in decreasing skin hydration and elasticity, wrinkle and pigment spot formation, and lead to premature skin aging (photo-aging) [11]. In recent years, there has been increasing interest in finding novel inhibitors of photo-aging process in the plant kingdom [4]. Due to the antioxidant and anti-inflammatory potency of bioactive constituents of the milk thistle (*S. marianum*) seeds, the extract SM and its polyphenols (flavonolignans) [12,13] seem to be suitable candidates for modulating processes associated with photo-aging. SM and its flavonolignans are not only effective radical scavengers, but they also form complexes with transition metals such as iron [14], thereby inhibiting the Fenton reaction. Previously we have also shown the regenerative potential of SM [15] and its pure flavonolignans SB and DHSB [16] on a UVA-damaged human keratinocyte line HaCaT. Newly we have demonstrated that SM and SB are able to prevent UVA-induced damage to normal human dermal fibroblasts [9]. The goal of this study was to evaluate the potential of SM and its pure flavonolignans to modulate the activity of enzymes participating in skin (photo)aging and to serve as active components of sunscreens to prevent to solar light-induced skin aging. 

In the tube tests, SM was found to be an effective inhibitor of collagenase, as its IC_50_ (2.03 ± 0.29 mg/L) was 3-fold lower than that of the standard inhibitor 1,10-phenantroline. SM also inhibited elastase activity, with a similar value of IC_50_ to the standard inhibitor oleanolic acid. In agreement with our results, Pientaweeratch et al. [17] recently also demonstrated that SM is an inhibitor of elastase. As for the pure flavonolignans, DHSB and SB were the most effective elastase inhibitors, but DHSB was much more effective (14-times) than SB. However, the amount of DHSB in the tested SM (see Table 1) is more than 100-fold lower than that of SB, so they both or together with other SM components, including the undefined polymeric fraction, participate in the anti-elastase effectiveness of SM. These results correlate with the antioxidant properties (radical scavenging activity) of SB and DHSB evaluated here by DPPH assay (Table 2). DHSB is the most effective substance of SM and more than 20-times as effective as SB. As for anti-collagenase activity, all the studied flavonolignans inhibit this enzyme activity, but significantly less effectively than SM. On the other hand, pure flavonolignans except for SD were more efficient than the standard inhibitor used, 1,10-Ph. Both the anti-collagenase and anti-elastase activity of SM and flavonolignans may be related to their ability to chelate metal ions. Recent study described potential of SB to make complexes with zinc that an essential component of MMPs [14,18]. The differences in collagenase inhibition between DHSB and the other flavonolignans were not as obvious as with elastase activity. Because no compound(s) tested showed dominating effect, all the flavonolignans present in SM probably participate in the anti-collagenase activity of SM; their effect could be additive and also influenced by their content in SM. The final inhibitory effect may be influenced by unidentified polymeric fraction as well. Although SM and flavonolignans were able to modulate elastase and collagenase activity and thus possibly protect components of the ECM, they all had no effect on the activity of hyaluronidase, which is responsible for decreases in skin hydration and wrinkle formation [19]. 

There are two basic mechanisms of sunscreen action to protect the skin against solar radiation. One is scattering the rays before entering the skin, typical of titanium and zinc oxide, the second is absorbing the rays [20]. Our simple evaluation (see Figure 2) showed that all the studied substances absorb in the UVB and UVA waveband and therefore can directly eliminate UV photons. In vitro photoprotective ability of SM and flavonolignans were expressed by sun protective factors (SPF_(290–320)_, SPF_(290–400)_ and UVA-PF), see Table 3. Within the UVB range (290–320 nm), the most abundant flavonolignan SB was the most potent component of SM with the SPF_(290–320)_ = 6.07 ± 0.19. The least effective flavonolignan in UVB range was DHSB (SPF_(290–320)_ = 3.64 ± 0.19). On the other hand, in long-wave part of UV spectrum, DHSB showed the highest potency with PF-UVA value of 2.90 ± 0.18. PF-UVA value of DHSB was double or nearly double compared to other flavonolignans and SM. Apart from the PF-UVA factor for DHSB, SM and the pure compounds were less effective than FA, used as a standard compound (Table 3). The concentration of SM and compounds in our evaluation of sun protective factors was rather small or lower (~0.024%, *w*/*v*) compared to practically used sunscreens concentrations in sun protective preparations (2–15%) (Directive EEC 76/768; Food and Drug Administration Sunscreen Monograph Final Rule). As showed in a previous study, SPF of SM increases with its concentration and sun protective ability may be significantly improved in combination with other sunscreens [21]. Thus, if SM and flavonolignans were used in a higher concentration, their protective effect would be much greater.

To conclude herein data, DHSB (the minor component of SM) was the most potent pure flavonolignan as it showed the highest UVA-PF value (Table 3), ability to scavenge DPPH radical (Table 2) and anti-collagenase and anti-elastase activity (Table 4). On other hand, DHSB photoprotective activity is limited by its instability, photodecomposition and the formation of reactive photodecomposition products and described phototoxicity [22]. Therefore, DHSB safety needs to be carefully evaluated in vivo or ex vivo. In contrast SM, the multicomponent standardized extract from the seed of *S. marianum* seems to be a suitable candidate for a component of dermatological preparations focusing on the prevention of sunlight-induced damage and (photo)aging. SM exhibited anti-collagenase and anti-elastase activities and was more effective than most of the pure flavonolignans. This is probably linked to the fact that SM is a mixture of several polyphenols that jointly participate and cooperate in SM’s action even when they are present in low amounts such as DHSB. The use of SM complex is also interesting for practical applications from the economical point of view, as extract preparation is easier and cheaper than the time-consuming and expensive isolation or synthesis of pure flavonolignans. Investigations of the (photo)protective and anti-(photo)aging potential as well as regenerative ability of SM and its flavonolignans at the cellular/tissue level will continue. 

## 4. Materials and Methods

### 4.1. Materials

Dimethyl sulfoxide (DMSO), elastase from human leukocytes, *N*-succinyl-Ala-Ala-Ala-*p*-nitroanilide, collagenase from *Clostridium histolyticum* (Type I), collagen-fluorescein, oleanolic acid, hyaluronidase from bovine testes (Type I-S), hyaluronic acid sodium salt, 4-(dimethylamino)benzaldehyde (DMAB), 1,10-phenanthroline (1,10-Ph), taxifolin, quercetin and other chemicals were from Sigma-Aldrich (Prague, Czech Republic). Methanol (HiPerSolv CHROMANORM for HPLC, LC-MS grade) was from VWR International s.r.o. (Stříbrná Skalice, Czech Republic). All solutions were prepared using reverse-osmosis deionized water (Ultrapur, Watrex, Prague, Czech Republic). Nitrogen and helium (99.999% for all) were obtained from Linde Gas (Prague, Czech Republic). 

### 4.2. Silymarin and its Flavonolignans

Silymarin (batch 17306S/089) containing 67.6% flavonolignans (see Table 1) and silybin (SB; batch 120692; purity 98%; mixture of diastereomers ca. 1:1) were kindly provided by IVAX Pharmaceuticals (Opava, Czech Republic). ISB (mixture of diastereomers ca. 95:5), SC (mixture of diastereomers ca. 9:1) and SD were isolated from SM [23] and DHSB was prepared by Gažák et al. [24]. Detailed SM analysis was performed according to Kosina et al. [25].

### 4.3. DPPH Scavenging Activity

Individual test compounds (0.01–2.5 g/L) in methanol (0.05 mL) were added to 0.1 mL of a methanolic solution of 2,2-diphenyl-1-picrylhydrazyl (DPPH; 20 mg/mL). After 30 min of incubation, the decrease in absorbance was measured at 517 nm. The determination was performed in triplicate and corrected to the blank sample (pure methanol). The radical scavenging activity of the tested compounds and SM was expressed as the concentration required for decoloration inhibition by 50% (IC_50_). The IC_50_ value for the pure compounds was expressed in both mass and molar concentration as mean ± SEM. For SM (a mixture of substances and an undefined fraction), the molar concentration was impossible to calculate. Quercetin was applied as a reference compound. 

### 4.4. Measurement of Spectra

Stock solutions of flavonolignans and SM (12.05 mg/L; DMSO) were diluted in PBS (pH 7.5). The blank sample contains the same aliquot of DMSO in PBS (0.5%). The absorption spectrum was scanned in a quartz cuvette against the blank in the range 250–500 nm using the fast speed scan in a UV-2401 PC UV-VIS spectrophotometer (Shimadzu, Kyoto, Japan).

### 4.5. Sun and UVA Protection Factors

The in vitro sun protection factors (SPF_(290–320)_ and SPF_(290–400)_) and UVA protective factor (PF-UVA) of DHSB, ISB, SC and SD were determined according to methods [26,27,28] As reference substance ferulic acid (FA) was used. [29]. The UV spectra of SM, SB, DHSB, ISB, SC and SD (24.1 mg/L; ethanol) and FA (50 µM; ethanol) were determined with a double-beam UV-VIS (Shimadzu UV-1800, Shimadzu, Kyoto, Japan) spectrophotometer in the range of 290–400 nm using a 1-cm quartz cell. 

The SPF_(290–320)_ was calculated using the following equation:

(1) $$SPF_{\left(290–320\right)}=CF\times \overset{320}{\underset{290}{\sum }}EE\left(\lambda \right)\times I\left(\lambda \right)\times Abs\left(\lambda \right)$$

where: *CF*–correction factor (10); *EE* (*λ*)–erythemal effect of spectrum, *I* (*λ*)–solar intensity spectrum, *Abs* (*λ*)–absorbance value at wavelength. The *EE* × *I* values were determined by Sayre et al. [26].

The SPF_(290–400)_ was calculated using the following equation:

(2) $$SPF_{\left(290–400\right)}=\frac{\sum_{290}^{400}S\left(\lambda \right)\times EA\left(\lambda \right)}{\sum_{290}^{400}S\left(\lambda \right)\times EA\left(\lambda \right)\times T\left(\lambda \right)}$$

where: *EA* (*λ*)–erythemal action spectrum, *S*–solar spectral irradiance, *T* (*λ*)–spectral transmittance value at the given wavelength. The *S* × *EA* values were determined by Diffey and Robson [27].

The PF-UVA was calculated using the following equation [27]:

(3) $$PF-UVA=\frac{\sum_{320}^{400}S\left(\lambda \right)\times EA\left(\lambda \right)}{\sum_{320}^{400}S\left(\lambda \right)\times EA\left(\lambda \right)\times T\left(\lambda \right)}$$

where: *EA* (*λ*), S and *T* (*λ*) are as above.

### 4.6. Anti-Aging Potential of Silymarin and Flavonolignans

The anti-aging potential of SM and its components was evaluated in a tube test as their ability to inhibit the activities of isolated hyaluronidase, collagenase and elastase. 

#### 4.6.1. Anti-Elastase Activity

Anti-elastase activity was evaluated using a method according to Ndlovu et al. [30] with small modifications. The reaction mixture contained 100 µL of 0.1 M HEPES buffer (pH 7.5), 10 µL of test sample/H_2_O/solvent/inhibitor, and 12.5 µL of elastase (1 U/mL) except for the blank. All tubes were incubated at room temperature for 5 min and the reaction was started by the addition of the substrate, *N*-methoxysuccinyl-Ala-Ala-Pro-Val-*p*-nitroanilide (10 µL, 4.4 mM; HEPES buffer; pH 7.5). The reaction was monitored as an increase in absorbance at 410 nm (ΔA/min) using a microplate spectrophotometer (INFINITE M200, Tecan Trading AG, Männedorf, Switzerland). Oleanolic acid was used as a standard inhibitor of the reaction. 

#### 4.6.2. Anti-Collagenase Activity

Anti-collagenase activity was measured by a method described by Maity et al. [31] with minor modifications. The reaction mixture contained 80 µL of buffer solution (0.05 M Tris-HCl, 0.15 M NaCl, 5 mM CaCl_2_, 0.2 mM sodium azide, pH 7.6), 10 µL of test sample/H_2_O/solvent/inhibitor and 10 µL of collagenase (200 U/mL) or water (blank for each sample). All tubes were incubated at room temperature for 5 min. To start the reaction, 20 µL of substrate (collagen-fluorescein, 0.6 mg/mL) was added. The reaction was monitored as the change in fluorescence at 495 nm (excitation) and 515 nm (emission) using a microplate spectrophotometer (INFINITE M200, Tecan Trading AG, Männedorf, Switzerland). 1,10-Ph was used as a standard inhibitor of the reaction. 

#### 4.6.3. Anti-Hyaluronidase Activity

Anti-hyaluronidase activity was determined using a method described by Ndlovu et al. [30] with minor modifications. The reaction mixture contained 25 µL of CaCl_2_ (12.5 mM), 12.5 µL of test sample/H_2_O/solvent/ inhibitor and 12.5 µL of hyaluronidase (1.5 mg/mL) except for the blank. All tubes were preheated (37 °C, 20 min) and then the substrate, hyaluronic acid (1 mg/mL in 0.1 M acetate buffer, pH 3.5) was added to start the reaction. After incubation (37 °C, 60 min), the reaction was stopped with 25 µL KBO_2_ (0.8 M) and tubes were heated briefly (100 °C, 3 min). Tubes were cooled to room temperature and 800 µL DMAB (4 g DMAB in 40 mL of acetic acid and 5 mL of 10 M HCl) was added. After incubation (20 min), samples were transferred onto a 96-well plate and fluorescence was measured at 545 nm (excitation) and 612 nm (emission) with a microplate spectrophotometer (INFINITE M200, Tecan Trading AG, Switzerland). Sodium aurothiomalate was used as a standard inhibitor.

### 4.7. Statistical Analysis 

The measurement of DPPH scavenging activity, sun, and UVA protection factor values and enzyme parameters was performed in triplicate in four independent experiments. Data were expressed as mean ± standard error of the mean (SEM). Statistical comparison was performed using Student’s *t*-test. Statistical significance was determined at *p* = 0.05.

## Figures and Tables

**Table 1:** Content of flavonolignans and taxifolin in silymarin, batch No. 17306S_089.

Compound	Retention Time (min)	Content (%)
SB A	6.41	16.34 ± 1.60
SB B	6.99	21.64 ± 1.53
ISB A	8.15	5.73 ± 1.16
ISB B	8.44	2.90 ± 0.65
SC A	3.10	13.73 ± 1.20
SC B	3.82	1.83 ± 0.15
SD	3.68	4.55 ± 0.62
DHSB	12.47	0.33 ± 0.07
DHSC	8.02	0.56 ± 0.09
TA	1.99	2.09 ± 0.41

Silybin A (SB A), silybin B (SB B), isosilybin A (ISB A), isosilybin B (ISB B), silychristin A (SC A), silychristin B (SC B), silydianin (SD), dehydrosilybin (DHSB), dehydrosilychristin (DHSC), taxifolin (TA). Results are expressed as mean ± standard error of the mean (SEM), *n* = 6.

**Table 2:** DPPH scavenging activity of silymarin and flavonolignans.

Compound	IC_50_ (µM)	IC_50_ (mg/L)
SB	527.86 ± 9.75 ^#,†^	254.43 ± 4.70 *^,†^
DHSB	26.25 ± 1.75 ^†^	12.60 ± 0.84 *^,†^
ISB	251.93 ± 6.56 ^#,†^	121.43 ± 3.16 *^,†^
SD	52.12 ± 1.93 ^#,†^	25.12 ± 0.93 ^†^
SC	38.05 ± 2.14 ^#,†^	18.34 ± 1.03 *^,†^
SM	-	25.38 ± 0.97 ^†^
QE	6.75 ± 0.87	2.04 ± 0.26

Silybin (SB), dehydrosilybin (DHSB), isosilybin (ISB), silydianin (SD), silychristin (SC), silymarin (SM), quercetin (QE). Data are expressed as mean ± SEM from four independent experiments. The IC_50_ (µM) of SM cannot be estimated. ^#^ Significantly different from DHSB at *p* = 0.05; * significantly different from SM at *p* = 0.05; ^†^ significantly different from QE at *p* = 0.05.

**Table 3:** Sun and UVA protection factors of silymarin and flavonolignans.

Compounds	SPF_(290–320)_	SPF_(290–400)_	UVA-PF
SM ^a^	5.50 ± 0.25 ^#^	2.49 ± 0.15 ^#^	1.52 ± 0.10 ^#^
SB ^a^	6.07 ± 0.19 ^#^	2.62 ± 0.16 ^#^	1.50 ± 0.11 ^#^
DHSB	3.64 ± 0.19 ^#^	2.38 ± 0.12 ^#^	2.90 ± 0.18
ISB	5.99 ± 0.11 ^#^	2.52 ± 0.14 ^#^	1.45 ± 0.13 ^#^
SD	4.35 ± 0.35 ^#^	2.01 ± 0.19 ^#^	1.31 ± 0.13 ^#^
SC	5.66 ± 0.14 ^#^	2.46 ± 0.16 ^#^	1.47 ± 0.14 ^#^
FA	7.51 ± 0.16	5.13 ± 0.28	3.36 ± 0.29

Silymarin (SM), silybin (SB), dehydrosilybin (DHSB), isosilybin (ISB), silydianin (SD), silychristin (SC). Ferulic acid (FA) was used as active component of sunscreens. ^a^ These values were published by Rajnochová Svobodová et al. 2018 [9]. The ethanolic solutions of studied compounds (50 μM) and SM (24.1 mg/L) were used. ^#^ Significantly different from FA at *p* = 0.05.

**Table 4:** Effect of flavonolignans and silymarin on elastase and collagenase activity.

Compounds	Elastase		Collagenase	
	IC_50_ (µM)	IC_50_ (mg/L)	IC_50_ (µM)	IC_50_ (mg/L)
SB	122.6 ± 4.8 ^#^	59.1 ± 2.3 *	52.2 ± 5.0 ^#^	25.2 ± 2.4 *
DHSB	8.6 ± 0.5 ^#^	4.1 ± 0.2 *	23.4 ± 2.9 ^#^	11.2 ± 1.4 *
ISB	~	~	50.8 ± 4.5 ^#^	24.5 ± 2.2 *
SD	~	~	190.3 ± 4.4 ^#^	91.8 ± 2.1 *
SC	~	~	95.9 ± 5.7 ^#^	46.3 ± 2.8 *
SM	-	6.27 ± 0.4	-	2.0 ± 0.3
OA	10.8 ± 0.6	4.9 ± 0.3 *	N.D.	N.D.
1,10-Ph	N.D.	N.D.	161.3 ± 4.6	29.1 ± 0.8 *

Silybin (SB), dehydrosilybin (DHSB), isosilybin (ISB), silydianin (SD), silychristin (SC), silymarin (SM). Oleanolic acid (OA) is a standard inhibitor of elastase. 1,10-Phenantroline (1,10-Ph) is a specific inhibitor of collagenase. ~ IC_50_ could not be determined due to the limited solubility of the studied compounds. N.D. IC_50_ was not determined for this compound. - SM is a mixture of substances, and the molar concentration of the extract is impossible to evaluate. ^#^ Significantly different from the respective standard inhibitor at *p* = 0.05; * significantly different from SM at *p* = 0.05.
